# Retinopathy and nephropathy in type 1 diabetes: role of HbA1c and blood pressure variability

**DOI:** 10.1007/s00592-025-02575-3

**Published:** 2025-09-17

**Authors:** Pavel Fatulla, Johnny Ludvigsson, Henrik Imberg, Thomas Nyström, Marcus Lind

**Affiliations:** 1 Department of Molecular and Clinical Medicine, Institute of Medicine, Sahlgrenska Academy, University of Gothenburg, Gothenburg, Sweden; 2 Department of Medicine, NU-Hospital Group, Trollhättan and Uddevalla, Sweden; 3 Crown Princess Victoria Children’s Hospital, Division of Paediatrics, Department of Biomedical and Clinical Sciences, Linköping University, Linköping, Sweden; 4 Statistiska Konsultgruppen Sweden, Gothenburg, Sweden; 5 Department of Clinical Science and Education, Internal Medicine, Södersjukhuset, Karolinska Institutet, Stockholm, Sweden; 6 Department of Medicine, Sahlgrenska University Hospital, Gothenburg, Sweden

**Keywords:** Type 1 diabetes, HbA1c variability, Blood pressure variability, Retinopathy, Nephropathy, Microvascular complications

## Abstract

**Aims:**

To examine the association between within-person variability in glycated hemoglobin A1c (HbA1c) and blood pressure (BP) with retinopathy and nephropathy in type 1 diabetes (T1D).

**Methods:**

This nationwide cohort included 9,358 individuals from the Swedish National Diabetes Register with T1D <5 years at inclusion (1998–2017) and ≥8 years follow-up. Variability in HbA1c, systolic BP (SBP), and diastolic BP (DBP) was calculated as updated SDs. Associations with microvascular complications were analyzed using logistic regression with generalized estimating equations, adjusted for demographic and clinical covariates.

**Results:**

Mean age at inclusion was 14.2 years, mean diabetes duration 1.2 years, and 44% were female. Over 10.7 years’ follow-up, retinopathy developed in 33% and nephropathy in 9.3%. SBP variability was significantly associated with pre-proliferative or proliferative retinopathy (aOR 1.13, 95% CI 1.00–1.27) and proliferative retinopathy/ laser photocoagulation (1.23, 1.04–1.45), as well as with any albuminuria (1.15, 1.08–1.23) and macroalbuminuria (1.29, 1.15–1.45). DBP variability was associated with any albuminuria (1.11, 1.03–1.19) and macroalbuminuria (1.28, 1.10–1.50). HbA1c variability was associated with any retinopathy (1.14, 1.08–1.20) and any albuminuria (1.12, 1.03–1.21).

**Conclusions:**

Beyond mean levels, higher variability in HbA1c and BP is associated with retinopathy and nephropathy. Stable BP control in patients with established retinopathy may be important to prevent progression to sight-threatening stages.

## Introduction

Poor long-term glycemic control is a well-established risk factor of microvascular complications in individuals with type 1 diabetes (T1D) [1]. Beyond mean glycated hemoglobin A1c (HbA1c) levels, within-person HbA1c variability has been proposed as an independent predictor of microvascular disease, including both retinopathy and nephropathy [2, 3]. Similarly, longitudinal variability in systolic blood pressure (SBP) has been linked to an increased risk of diabetic nephropathy [4]. This study aimed to evaluate whether variability in HbA1c and blood pressure is associated with the development of retinopathy and nephropathy—two major contributors to morbidity and mortality in individuals with T1D.

## Methods

This registry-based cohort study, previously described in detail [1, 5], included individuals with T1D of less than five years’ duration, registered in the Swedish National Diabetes Register between 1998 and 2017. Eligible participants had at least four clinical visits with complete data on risk factors and outcomes, and a minimum of eight years of follow-up from diabetes onset.

To account for differences in diabetes duration and the potentially varying influence of risk factors over time, participants were grouped into subcohorts based on years since diagnosis: 8–9, 10–11, 12–13, 14–15, and 16–20 years, with a maximum follow-up time of 20 years.

Variability in HbA1c, SBP, and diastolic blood pressure (DBP) was calculated using the updated standard deviation (SD) of available measurements during follow-up. Retinopathy was classified as (i) any retinopathy, (ii) pre-proliferative diabetic retinopathy or proliferative retinopathy; and (iii) proliferative retinopathy/laser photocoagulation. Nephropathy was defined as the presence of microalbuminuria or macroalbuminuria. The study was approved by the Swedish Ethical Review Authority.

Associations between risk factor variability and outcomes were assessed using logistic regression with generalized estimating equations (GEE), accounting for repeated measures. Models were first adjusted for age and sex, then additionally for clinical covariates. All predictors were standardized to Z-scores, and results presented as adjusted odds ratios (aORs) with 95% confidence intervals (CIs) per 1 Z-score increase in each risk factor.

## Results

**Fig. 1:**
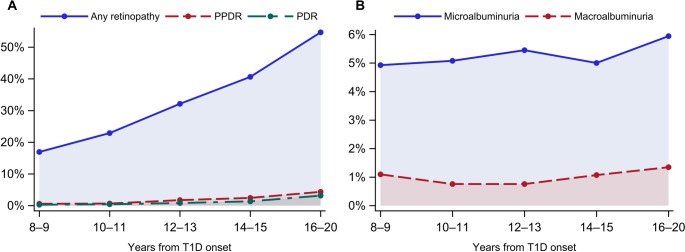
Prevalence of retinopathy (A) and nephropathy (B) by years since type 1 diabetes (T1D) onset. PPDR = pre-proliferative diabetic retinopathy, PDR = proliferative diabetic retinopathy

A total of 9,358 individuals with T1D were included in the study. At inclusion, mean (SD) age was 14.2 (7.8) years, and diabetes duration was 1.2 (1.6) years; 44% were female. Over a mean follow-up period of 10.7 (3.2) years, the average HbA1c was 7.9% (1.0%) or 63 (11) mmol/mol; mean SBP was 116 (8.5) mm Hg, and DBP 69 (5.8) mm Hg. Overall, 33% of participants developed retinopathy and 9.3% nephropathy **(**Fig. 1**)**.

SBP variability, but not HbA1c variability, was associated with both pre-proliferative and proliferative retinopathy. In contrast, for any retinopathy HbA1c-variability showed an association, as was the case for any albuminuria. Blood pressure variability was significantly associated with any albuminuria as well as with macroalbuminuria **(**Table 1**)**.

**Table 1:** Risk factors for diabetic retinopathy and nephropathy in T1D, expressed as odds ratios (ORs) per 1 SD increase in HbA1c, SBP, and DBP variability

Outcome / Risk factor	Per x units increase	Model 1, aOR (95% CI)^a^	*P* value	Model 2, aOR (95% CI)^b^	*P* value
Any retinopathy^c^					
HbA1c SD	per 4.7 mmol/mol increase	1.48 (1.42, 1.54)	< 0.001	1.14 (1.08, 1.20)	< 0.001
SBP SD	per 3.2 mm Hg increase	1.04 (1.00, 1.09)	0.038	1.00 (0.96, 1.04)	0.91
DBP SD	per 2.3 mm Hg increase	1.08 (1.03, 1.12)	< 0.001	1.03 (0.99, 1.07)	0.16
Pre-proliferative or proliferative retinopathy ^d^					
HbA1c SD	per 4.7 mmol/mol increase	1.79 (1.63, 1.95)	< 0.001	1.01 (0.89, 1.14)	0.90
SBP SD	per 3.2 mm Hg increase	1.21 (1.09, 1.35)	< 0.001	1.13 (1.00, 1.27)	0.041
DBP SD	per 2.3 mm Hg increase	1.08 (0.96, 1.21)	0.20	0.98 (0.87, 1.11)	0.75
Proliferative retinopathy or laser photocoagulation of the retina^e^					
HbA1c SD	per 4.7 mmol/mol increase	1.83 (1.60, 2.09)	< 0.001	1.08 (0.92, 1.28)	0.35
SBP SD	per 3.2 mm Hg increase	1.30 (1.13, 1.51)	< 0.001	1.23 (1.04, 1.45)	0.014
DBP SD	per 2.3 mm Hg increase	1.18 (1.01, 1.38)	0.040	1.07 (0.89, 1.28)	0.46
Any albuminuria^f^					
HbA1c SD	per 4.7 mmol/mol increase	1.40 (1.32, 1.48)	< 0.001	1.12 (1.03, 1.21)	< 0.001
SBP SD	per 3.2 mm Hg increase	1.20 (1.13, 1.28)	< 0.001	1.15 (1.08, 1.23)	< 0.001
DBP SD	per 2.3 mm Hg increase	1.16 (1.08, 1.25)	< 0.001	1.11 (1.03, 1.19)	0.005
Macroalbuminuria^g^					
HbA1c SD	per 4.7 mmol/mol increase	1.43 (1.27, 1.61)	< 0.001	1.13 (0.96, 1.33)	0.15
SBP SD	per 3.2 mm Hg increase	1.34 (1.20, 1.50)	< 0.001	1.29 (1.15, 1.45)	< 0.001
DBP SD	per 2.3 mm Hg increase	1.32 (1.14, 1.53)	< 0.001	1.28 (1.10, 1.50)	0.002

Statistical analyses were performed using logistic regression with generalized estimating equations (GEE) to account for repeated measurements from the same individuals across different subcohorts (8–9, 10–11, 12–13, 14–15, and 16–20 years after T1D diagnosis). Risk factors were standardized to Z-scores, and results are reported as adjusted odds ratios (aORs) with 95% confidence intervals (CIs) per 1 Z-score (i.e., *x* units) increase

^a^ Model 1: Adjusted for age and sex

^b^ Model 2: For HbA1c variability, additionally adjusted for HbA1c area under the curve (AUC), mean SBP, DBP, BMI, LDL, HDL, triglycerides, total cholesterol, and smoking status. For SBP and DBP variability, additionally adjusted for HbA1c AUC and corresponding mean blood pressure

^c^ Any retinopathy (simplex, PPDR, PDR, or laser photocoagulation) vs. no retinopathy

^d^ Pre-proliferative retinopathy or proliferative retinopathy (PPDR, PDR, or laser photocoagulation) vs. simplex retinopathy or no retinopathy

^e^ Proliferative retinopathy or laser photocoagulation vs. less severe forms of retinopathy or no retinopathy

^f^ Any albuminuria (micro- or macroalbuminuria) vs. no albuminuria

^g^ Macroalbuminuria vs. microalbuminuria or no albuminuria

Abbreviations: aOR, adjusted odds ratio; AUC, area under the curve; BMI, body mass index; CI, confidence interval; DBP, diastolic blood pressure; GEE, generalized estimating equations; HbA1c, glycated hemoglobin A1c; HDL, high-density lipoprotein; LDL, low-density lipoprotein; OR, odds ratio; PDR, proliferative diabetic retinopathy; PPDR, pre-proliferative diabetic retinopathy; SBP, systolic blood pressure; SD, standard deviation; T1D, type 1 diabetes

## Discussion

In this population-based nationwide study of persons with T1D, high SBP variability significantly increased the risk for progression to severeforms of retinopathy, being potentially sightthreatening. Moreover, blood pressure variability increased the risk for both early and more advanced stages of nephropathy, whereas HbA1c-variability was mainly associated with milder stages of retinopathy and nephropathy.

The association between SBP variability and advanced retinopathy may reflect increased vascular vulnerability in severe stages of disease, where microvascular damage is already established. While SBP variability has previously been linked to nephropathy [4], our results suggest that it may also contribute to the development of advanced retinal complications. Our findings may be explained by intermittent vascular stress due to SBP fluctuations, which may trigger oxidative stress and promote endothelial dysfunction. In the retina and kidneys, organs sensitive to hemodynamic changes, such variability could accelerate the progression of pre-existing microvascular injury. These hypotheses align with previous studies suggesting that not only absolute blood pressure levels, but also their stability over time, influence vascular outcomes. Although medication data were not available in the current study, it would be of interest for future research to investigate whether pharmacological agents with both antihypertensive and organ-protective properties could reduce the risk associated with blood pressure variability in individuals with type 1 diabetes.

In this study our findings highlight the need for an increased focus in clinical practice, when significant retinopathic lesions have emerged, to keep a well-balanced and stable blood pressure in order to prevent progression to advanced sight-threatening retinopathy.

A strength of this study is the population-based design following persons with T1D from diagnosis and onwards over 8–20 years with repeated measures of risk factors. Limitations include the absence of continuous glucose monitoring (CGM) data. Another limitation is the irregular timing of clinical measurements, which may affect the estimation of HbA1c and blood pressure variability, as well as the ability to determine the duration of exposure to elevated levels.

In conclusion, besides the overall mean levels of HbA1c and blood pressurea, a high variability in these risk factors further contributes to development of retinopathy and nephropathy. Obtaining a stable and well-balanced blood pressure in patients with established retinopathy needs attention in persons with T1D in clinical practice. Avoiding peaks and periods with high blood pressure is likely essential to prevent vulnerable lesions of retinopathy to progress to sight-threatening stages.
